# Error in Results

**DOI:** 10.1001/jamanetworkopen.2026.44287

**Published:** 2026-10-16

In the Original Investigation titled “Physicians’ Experiences With Life-Threatening Illness and Return to Work: A Qualitative Study,”^1^ published September 9, 2026, there was an error in the Results. The 22 physicians were from 8, not 7, specialties. Pathology and laboratory medicine was inadvertently omitted from the list. This article has been corrected.^1^
